# Oral mobility reflects rate of progression in advanced Friedreich’s ataxia

**DOI:** 10.1002/acn3.50879

**Published:** 2019-08-25

**Authors:** Stéphanie Borel, Peggy Gatignol, Mustapha Smail, Marie‐Lorraine Monin, Claire Ewenczyk, Didier Bouccara, Alexandra Durr

**Affiliations:** ^1^ Sorbonne Université, INSERM, UMRS1159 Réhabilitation Chirurgicale Mini‐Invasive et Robotisée de l’Audition Paris France; ^2^ AP‐HP, Service d’ORL, Hôpital Universitaire Pitié-Salpêtrière Paris France; ^3^ Sorbonne Université, INSERM, UMRS1158 Neurophysiologie Respiratoire Expérimentale et Clinique Paris France; ^4^ Sorbonne Université, Institut du Cerveau et de la Moelle épinière (ICM), AP‐HP, INSERM, CNRS, Hôpital Universitaire Pitié-Salpêtrière Paris France

## Abstract

Our objective was to identify a sensitive marker of disease progression in Friedreich’s ataxia. We prospectively evaluated speech, voice, and oromotor function in 40 patients at two timepoints. The mean disease duration was 20.8 ± 9.8 years and mean SARA score 23.7 ± 8.6 at baseline. Oral motor mobility, assessed by a combination of movements of the face, eyes, cheeks, lips, and tongue, decreased significantly after 1 year (*P* < 0.0001). The standardized response mean over 12 months was considered as large for oral mobility (1.26) but small for SARA (0.12). Oral mobility could therefore be a sensitive marker in therapeutic trials.

## Introduction

Friedreich ataxia (FA) is an inherited autosomal recessive mitochondrial disorder and the most frequent inherited ataxia in Europe. It is characterized by sensory and cerebellar involvement, pyramidal signs, muscle weakness, optic atrophy, and extra‐neurological signs, such as cardiomyopathy and diabetes.^1^ FA starts in adolescence and FA adults are wheelchair bound at a mean age of 25 years.^1^ Significant increases in ataxia, evaluated by the SARA (Scale for the Assessment and Rating of Ataxia), are observed less in the most severely affected patients, indicating a ceiling effect.^2^ Later‐stage patients are often too severely impaired to be assessed using most measures of arm and leg function and the measurement of dysarthria has been suggested to be useful for annual follow‐up of changes but has not yet been tested longitudinally.^3^ Dysarthria in FA has been well described.^4^, ^5^, ^6^ Changes of speech and voice have either been shown to be significant after 2 years^7^ or not after 1 or 2 years of follow‐up.^8^. We aimed to identify a sensitive follow‐up marker to detect changes in voice and speech over 1 year amenable for advanced FA. We studied speech, voice and oral mobility in FA, and evaluated their auditory function known to be affected.^1^, ^9^, ^10^ Thus, we prospectively investigated the decline in voice, speech, and hearing of 40 FA patients with a battery of tests over 1 year.

## Methods

Forty FA patients were enrolled at the Pitié‐Salpêtrière University Hospital in Paris into a prospective, longitudinal evaluation of hearing, speech, and voice (UE FP7‐HEALTH‐2009/contract n°E10015DD) within the EFACTS framework (www.e-facts.eu, NCT02069509). The study was accepted by the regional health institutional review board IDF‐6 on February 20, 2015. Informed consent was obtained from all individual participants included in the study.

Neurological evaluation included SARA, a semi‐quantitative scale developed to assess ataxia, with values from 0 (no ataxia) to 40 (most severe ataxia). Eight items assess stance, sitting, speech disturbances, finger chase, dysmetria, nose–finger test, tremor, fast alternating hand movements, and heel–shin slide.^2^, ^11^ In addition, we used the CCFS (composite cerebellar functional score), a quantitative assessment including a nine‐hole pegboard test and the click test (time needed to perform 10 finger‐pointing cycles).^12^

In order to evaluate the auditory function, the patients underwent pure tone audiometry. The average of the thresholds at 500, 1000, 2000, and 4000 Hz and the percentage of correct repetition of dissyllabic words in silence were calculated.

The mobility of lingual and facial muscles involved in articulation, mimicry, and oral mobilization during the first stage of swallowing, was evaluated by computerized evaluation: “Bucco‐lingual‐facial motility” (MBLF) (ADEPRIO, edition 2011). Photographs of several articulatory positions were shown to the patients and they were instructed to imitate 37 movements (Fig. S1). Each item was scored on a scale from 0 (maximal impairment) to 3 (optimal). At least 37 items across five domains that involve the use of the three cranial nerves, VII, X, and XII, were tested (Table S1). The total MBLF score is derived by summing each category and ranges from 0 to 111 (worst to best). A profile of the scores is thus obtained by domain (face: /6, eye: /9, cheeks: /30, lips: /27, and tongue: /39, Fig. S2). Speech intelligibility was evaluated by the French TPI test from the BECD battery (Ortho Éditions, 2006), an identification task consisting of a multiple choice for 13 sets of four words representative of 13 phonetic contrasts, with a maximum possible score of 52. Acoustic analysis was based on MonPaGe, a computerized protocol for the evaluation of pathological speech in French,^13^ and Praat software (http://www.fon.hum.uva.nl/praat/, version 5.3.40). The oral diadochokinetic (DDK) was assessed by the repetition of the syllables [badego] as fast and accurately as possible in a single breath. [badego], such as [pata] or [pataka], is a test of articulatory diadochokinesis that measures "sequential motion rate". Compared to [pataka], [badego] associates to each consonant a vowel with the same place of articulation, to facilitate articulation. Furthermore, in [badego] all phonemes are voiced by the vibration of the vocal folds. Thus, the patient does not need to alternate voicing movement between the voiced vowel and the voiceless consonant, contrary to [pata]. Therefore [badego] sequence is probably slightly easier to pronounce than [pataka]. The number of syllables correctly repeated over the first 4 sec was measured. Speech rate was measured using the short four‐syllable sentence “Laurie l’a lu.” (“Laurie read it”).^13^ For the Voice assessment, each subject was asked to produce the sustained vowel [a] for 3 sec. The harmonics‐to‐noise ratio (HNR) is a measure in dB that quantifies the amount of noise in the voice. The fundamental frequency (F0, in Hertz) was measured. Jitter and shimmer (in %) were used to determine the perturbation index of the laryngeal vibrator. The maximum phonation time (MPT) was measured as the longest period during which the patient can sustain the phonation of the vowel [a] in a single breath. "Voice Handicap index" (VHI) was used to evaluate the handicap related to speech and voice disabilities,^14^, ^15^ with a total score from zero to 120 (highest perceived handicap) and from 0 to 40 for each of its three subscales Emotional, Functional, and Physical.

All statistical analyses were performed using Statview 5.0.1 (SAS Institute Inc.). Audiological, and correlations were done, at baseline, using the Pearson test. Results at baseline were compared to those at the follow‐up visit with paired t‐tests. Because the variables were probably dependent on each other, Bonferroni corrections for multiple analyses were applied. Thus, *P* < 0.0031 was considered to be significant for comparisons and *P* < 0.00071 for correlations. In order to assess how measures changed over 1 year, research participants were assessed at two timepoints and we used standardized response mean (SRM) as one type of effect size measure^16^ by the formula [mean at baseline – mean at follow‐up/SD at baseline]. According to Husted et al. and others, values of 0.20, 0.50, and 0.80 or greater have been proposed to represent small, moderate, and large responsiveness, respectively.^16^

## Results

The study group consisted of 20 women and 20 men, aged 38.6 ± 11.7 years, ranging from 22 to 69 at examination. The mean age at onset was 17.8 ± 9.4 years, ranging from 3 to 46. The mean number of GAA repeats on the smaller allele was 472, ranging from 80 up to 850, and on the larger 761, ranging from 166 to 1200. Two patients had point mutations in one of the two alleles. A wheelchair was used by 26 of 40 (65%). One patient withdrew consent for the follow‐up visit and one could not complete the follow‐up because the handicap became too advanced. At baseline 3 of 39 patients had a moderate hearing impairment in at least one ear and 8 of 39 were mildly hearing‐impaired; two wore hearing aids. All but one patient achieved 100% intelligibility on the vocal audiometry test. After 1 year, no additional patients became hearing‐impaired or required a hearing aid and audiological levels did not decrease. There was no correlation between the severity of hearing impairment and voice scores. SARA increased significantly over time, but not the CCFS (Table 1). The total oral motor mobility score decreased significantly after 1 year (*P* = 0.0002). Among the FA patients with normal MBLF scores at baseline (18/36), most (11/18) worsened over 1 year, as well as those who started with impaired oromotor function (9/18). Mobility of the tongue decreased most significantly (*P* = 0.00018), whereas the mobility of the lips and cheeks remained stable. Furthermore, at baseline, the MBLF score correlated with disease severity, measured by the SARA (*r* = −0.63 *P* < 0.0001) and CCFS (*r* = 0.67 *P* < 0.0001) as well as with VHI‐Functional subscale (*r* = −0.53 *P* = 0.0004) and diadochokinesis (*r* = 0.71 *P* < 0.0001). But there was no correlation between the aggravation of MBLF and the aggravation of the SARA, the CCFS, the VHI or the DDK. The intelligibility score (TPI), fundamental frequency, HNR, jitter, shimmer, and VHI did not decrease over 1 year. The syllabic rate measured in the DDK task correlated with the SARA at baseline (*r* = −0.58 *P* = 0.0001) but remained stable over one year, as well as the rate of the production of a short sentence. The maximum phonation time was shorter for an increasing number of GAA1 repeats (*r* = −0.63, *P* < 0.0001), and SARA score (*r* = −0.55 *P* = 0.0004). However, the MPT remained stable over 1 year. Standardized Response Mean was large for MBLF (1.26), but small for SARA (0.12). No gender effect was found in the results for SARA or MBLF, neither for the score at baseline, nor for the delta between baseline and follow‐up scores (unpaired *t*‐test).

**Table 1:** Audiological, speech, and voice evaluation at baseline and follow‐up visits.

	*N*	Baseline	Follow‐up	Paired *t*‐test
		Mean ± SD [min–max]	Mean ± SD [min‐max]	*P*‐value
Neurological evaluation				
SARA^1^ (/40)	35	23.66 ± 8.60 [9–38]	24.70 ± 8.77 [8, 5–38]	0.003*
CCFS^2^	29	1.234 ± 0.144 [1.048–1.544]	1.269 ± 0.166 [1.065–1.656]	0.94
Audiometric evaluation				
R: PTA^3^ (dB^4^)	36	14.8 ± 10.44 [0–45]	15.5 ± 8.96 [4.3–38.6]	0.33
L: PTA^3^ (dB^4^)	36	15.8 ± 10.39 [0–45]	17.6 ± 10.28 [2.1–44.2]	0.33
R: Speech audio. (%)	36	98.9 ± 6.67 [60–100]	99.72 ± 1.67 [90–100]	0.33
L: Speech audio. (%)	36	98.9 ± 6.67 [60–100]	99.72 ± 1.67 [90–100]	0.48
Oromotor function				
MBLF^5^ (/111)	36	109.1 ± 2.69 |101–111]	105.7 ± 4.49 [95–111]	0.0002*
Evaluation of speech and voice				
VHI^6^ (/120)	36	33.9 ± 17.27 [6–66]	34.9 ± 18.52 [4–62]	0.52
TPI^7^ (/52)	35	50.5 ± 1.72 [46–52]	50.1 ± 1.86 [46–52]	0.14
MPT^8^ (sec)	32	12.2 ± 4.24 [5–21]	11.8 ± 5.20 [4–24]	0.43
Acoustical analysis				
*F*_0_ 9 (Hz)	33	159.2 ± 40.73 [102–270]	158.5 ± 39.97 [93–240]	0.18
Jitter (%)	33	0.82 ± 0.77 [0.29–4.53]	0.89 ± 0.87 [0.29–4.17]	0.76
Shimmer (%)	31	2.98 ± 1.58 [0.98–7.3]	3.22 ± 2.75 [0.62–14.21]	0.77
HNR^9^ (dB^3^)	30	22.3 ± 3.65 [13.7–27.5]	20.41 ± 374 [131–26.2]	0.09
Speech rate (syllable/sec)				
Reading sentence	33	3.56 ± 0.83 [2.23–5.58]	3.65 ± 0.75 [2.28–4.99]	0.39
Diadochokinesis [badego]	35	4.11 ± 0.80 [2.5–6]	3.81 ± 0.74 [2.25–5.25]	0.007

R, right; L, left. Speech audio: speech audiometry.

1 Scale for the assessment and rating of ataxias.

2 Composite cerebellar functional score.

3 Pure tone average.

4 Decibel.

5 Mobility bucco‐linguo‐facial.

6 Voice handicap index.

7 Phonetic test of intelligibility.

8 Maximum phonation time.

9 Fundamental frequency

10 Harmonic‐to‐noise‐ratio.

* Significant after Bonferroni correction.

## Discussion

In this prospective longitudinal study, we found significant changes in oromotor function after 1 year in FA, showing that oral mobility can reflect the rate of progression of the disease. Evaluation of the SARA in a prospective European cohort showed an increase of 0.77 points per year, but significantly less in FA patients with a SARA above 30,^17^ showing a ceiling effect for this scale. Later‐stage FA patients are frequently too severely impaired to be assessed by most measures.^3^ In our study, the SARA increased as expected,^2^, ^17^ but the Standardized Response Mean of this scales was small, while the SRM of oral mobility measures was large. Oromotor function has already been reported to correlate with the severity of ataxia in FA patients^3^, ^18^, ^19^ and voluntary lingual movements were specifically reported to be impaired,^19^, ^20^ but the Standardized Response Mean of these measures had previously not been studied. Significant changes after 2 years in speech and voice have been shown in FA patients, but oromotor function has never been studied longitudinally.^4^ We and others^5^ showed that measures of speech and voice remain stable over 1 year, in contrast to oromotor function, which changed significantly after only 1 year. Speech and voice tests are possibly compensated by adjustments during speech by the patient and are therefore less sensitive. In contrast, during a simple task of articulatory mobility, there are less possibilities of compensating. We controlled for confounding variables by assessing hearing. Sensorineural hearing‐loss was evident for 28% of patients, more than previously reported^1^, ^21^, ^22^ except one study.^11^ Nevertheless, hearing impairment did not influence voice scores, allowing us to consider all patients. Our study was limited by the fact that we included adult FA consisting of patients with advanced disease after more than 20 years of progression. Even if therapeutic approaches will probably be tested in early stage patients because the probability of reversion is expected to be higher, if some therapy can be used to alleviate disease burden even in later stages, it is important to evaluate its effect. Oromotor measurement was feasible, even in advanced FA, as the test took less than 5 min. Furthermore, it can be used independently of language or age. In addition, oromotor function is a good indicator of functional vocal handicap. Further studies are needed to assess the responsiveness of oromotor function in earlier stages of the disease.

## Author Contributions

SB: Design and conceptualization of the study, analysis and interpretation of the data, and drafting and revision of the manuscript for intellectual content.

PG: Design and conceptualization of the study, analysis and interpretation of the data, and drafting and revision of the manuscript for intellectual content.

MS: Contributor and revision of the manuscript for intellectual content.

MLM: Contributor and revision of the manuscript for intellectual content.

CE: Contributor and revision of the manuscript for intellectual content.

DB: Design and conceptualization of the study and revising of the manuscript for intellectual content.

AD: Design and conceptualization of the study, analysis and interpretation of the data, and drafting and revision of the manuscript for intellectual content.

## Conflict of Interest

Stéphanie Borel: None; Peggy Gatignol: None; Mustapha Smail: None; Marie‐Lorraine Monin: None; Claire Ewenczyk: None; Didier Bouccara: None; Alexandra Durr: None.

## Supporting information


**Figure S1.** Examples of photographs. Left: Close your eyes (Orbicularis oculi); Right: Pinch your lips (Compressor/buccinator).

**Figure S2.** Example of results for face, eye, lips, jaw and mandible, and tongue movements. To compare, the standardized means of 108 control subjects aged from 20 to 79 years with sex ratio 1:1 and distributed among three different education levels are represented as “M” on the graph.

**Table S1.** Items (translated from French to English by Gatignol et Lannadère).
