# Supplementary figures and images for: Oral mobility reflects rate of progression in advanced Friedreich’s ataxia
Source: Ann Clin Transl Neurol. 2019 Aug 25;6(9):1888–92. doi: 10.1002/acn3.50879 (PMC6764486; doi:10.1002/acn3.50879)

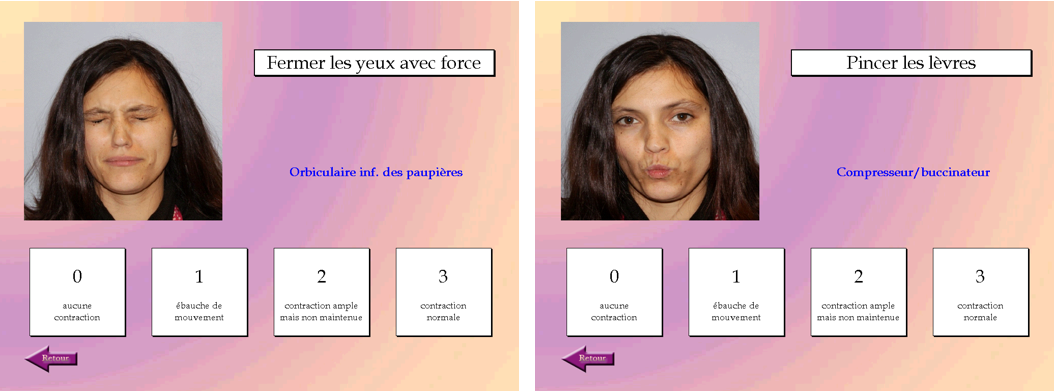

Supplement: Supplementary file 1 — Figure S1. Examples of photographs. Left: Close your eyes (Orbicularis oculi); Right: Pinch your lips (Compressor/buccinator). [file ACN3-6-1888-s001.PNG]

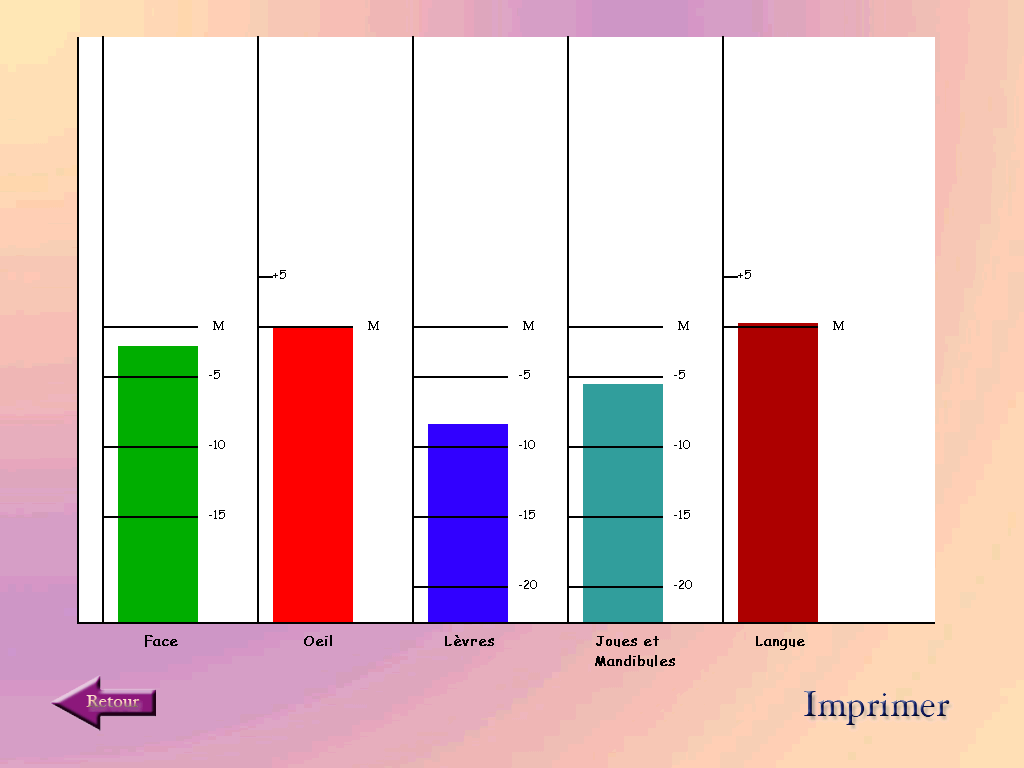

Supplement: Supplementary file 2 — Figure S2. Example of results for face, eye, lips, jaw and mandible, and tongue movements. To compare, the standardized means of 108 control subjects aged from 20 to 79 years with sex ratio 1:1 and distributed among three different education levels are represented as “M” on the graph. [file ACN3-6-1888-s002.png]
