# Supplementary material for: Oral mobility reflects rate of progression in advanced Friedreich’s ataxia
Source: Ann Clin Transl Neurol. 2019 Aug 25;6(9):1888–92. doi: 10.1002/acn3.50879 (PMC6764486; doi:10.1002/acn3.50879)
Supplement: Supplementary file 3 — Table S1. Items (translated from French to English by Gatignol et Lannadère). [file ACN3-6-1888-s003.doc]

Supplemental Table 1: Facial and Lingual Motor Skills Assessment (Motricité bucco-linguo facial/MBLF), developed and validated in french language by Gatignol et Lannadère, Adeprio editor, 2011, France, available at <https://adeprio.com/eurl/produit/mblf-adultes/>. Items (translated from French to English by Gatignol et Lannadère).

| **Facial areas** | **Oral Motor Tasks** | **Muscles** | **0** | **1** | **2** | **3** | **Score** |
| --- | --- | --- | --- | --- | --- | --- | --- |
| **Face**  **/6** | Symmetry at rest |  |  |  |  |  | **/3** |
|  | Symmetry when smiling |  |  |  |  |  | **/3** |
| **Eyes**  **/9** | Close your eyes | Orbicularis oculi |  |  |  |  | **/3** |
|  | Raise your eyebrows | Occipito-frontalis |  |  |  |  | **/3** |
|  | Frown | Corrugator supercilii |  |  |  |  | **/3** |
| **Lips**  **/27** | Pinch your lips | Compressor/buccinator |  |  |  |  | **/3** |
|  | Stretch your lips | Zygomaticus/risorius |  |  |  |  | **/3** |
|  | Keep lips closed strongly | Orbicularis/masseter |  |  |  |  | **/3** |
|  | Open mouth smile | Zygomaticus/risorius |  |  |  |  | **/3** |
|  | Show the upper teeth | Levator labii superioris |  |  |  |  | **/3** |
|  | Show the lower teeth | Mentalis |  |  |  |  | **/3** |
|  | Say « u » | Orbicularis oris |  |  |  |  | **/3** |
|  | Whistle | Orbicularis oris |  |  |  |  | **/3** |
|  | Blow | Orbicularis oris |  |  |  |  | **/3** |
| **Cheeks and jaws**  **/30** | Open your mouth | Buccinator/orbicularis oris |  |  |  |  | **/3** |
|  | Close your mouth | Masseter/orbicularis oris |  |  |  |  | **/3** |
|  | Puff off the cheeks | Buccinator/orbicularis oris |  |  |  |  | **/3** |
|  | Puff left cheek | Buccinator/orbicularis oris |  |  |  |  | **/3** |
|  | Puff right cheek | Buccinator/orbicular |  |  |  |  | **/3** |
|  | Pass the air from one cheek to another | Buccinator/orbicularis oris |  |  |  |  | **/3** |
|  | Suck in the cheeks | Buccinator/orbicularis oris |  |  |  |  | **/3** |
|  | Left jaw open mouth | Pterygoid |  |  |  |  | **/3** |
|  | Right jaw open mouth | Pterygoid |  |  |  |  | **/3** |
|  | Chew closed mouth |  |  |  |  |  | **/3** |
| **Tongue**  **/39** | Stick the tongue out | Genioglossus/transverse muscle |  |  |  |  | **/3** |
|  | Bring in the tongue | Hyoglossus/superior longitudinal muscle |  |  |  |  | **/3** |
|  | Put the tongue to the right corner of the mouth | Pharyngoglossus |  |  |  |  | **/3** |
|  | Put the tongue to the left corner of the mouth | Pharyngoglossus |  |  |  |  | **/3** |
|  | Put it on top | Superior longitudinal muscle |  |  |  |  | **/3** |
|  | Put it down | Superior longitudinal muscle |  |  |  |  | **/3** |
|  | Put your tongue on your teeth | Styloglossus/hyoglossus |  |  |  |  | **/3** |
|  | Move the tongue inside the right cheek |  |  |  |  |  | **/3** |
|  | Move the tongue inside the left cheek |  |  |  |  |  | **/3** |
|  | Raise the tip in the mouth | Pharyngoglossus |  |  |  |  | **/3** |
|  | Raise the tip out of the mouth | Styloglossus |  |  |  |  | **/3** |
|  | Click of the disapproval/disagreement | Styloglossus |  |  |  |  | **/3** |
|  | Rhythm of galloping horse | Styloglossus |  |  |  |  | **/3** |
|  | **TOTAL** |  |  |  |  |  | **/111** |

Footnotes

Rating: 0 = no contraction; 1 = initiated movement; 2 = almost complete movement; 3 = normal contraction. Face symmetry: 0 = severe/complete asymmetry; 1 = significant/moderate asymmetry; 2 = mild asymmetry; 3 = no asymmetry
